# Contribution of Quercetin to the Composition and Antioxidant Properties of *Monascus* Exopolysaccharides

**DOI:** 10.3390/foods12051004

**Published:** 2023-02-27

**Authors:** Haiyun Yang, Hui Meng, Liuming Xie, Zhibing Huang

**Affiliations:** 1 State Key Laboratory of Food Science and Technology, Nanchang University, No. 235 Nanjing East Road, Nanchang 330047, China; 2 Sino–German Joint Research Institute, Nanchang University, No. 235 Nanjing East Road, Nanchang 330047, China

**Keywords:** *Monascus*, exopolysaccharides, medium composition, culture conditions, antioxidant capacity

## Abstract

Exopolysaccharides are important metabolites of *Monascus* with healthy activities. However, the low production level limits their applications. Hence, the aim of this work was to increase the yield of exopolysaccharides (EPS) and optimize liquid fermentation by adding flavonoids. The EPS yield was optimized via both medium composition and culture conditions. The optional fermentation conditions achieved for EPS production of 7.018 g/L were 50 g/L sucrose, 3.5 g/L yeast extract, 1.0 g/L MgSO_4_·7H_2_O, 0.9 g/L KH_2_PO_4_, 1.8 g/L K_2_HPO_4_·3H_2_O, 1 g/L quercetin, and 2 mL/L Tween-80, with pH 5.5, inoculum size 9%, seed age 52 h, shaking speed 180 rpm, and fermentation culture 100 h, respectively. Furthermore, the addition of quercetin increased EPS production by 11.66%. The results also showed little citrinin residue in the EPS. The exopolysaccharides’ composition and antioxidant capacity of quercetin-modified exopolysaccharides were then preliminarily investigated. The addition of quercetin changed the composition of the exopolysaccharides and the molecular weight (*Mw*). In addition, the antioxidant activity of *Monascus* exopolysaccharides was monitored using 2,2-diphenyl-1-picrylhydrazyl (DPPH), 2,2′-Azinobis-(3-ethylbenzthiazoline-6-sulphonate) (ABTS^+^), and -OH. *Monascus* exopolysaccharides have good scavenging ability of DPPH and -OH. Furthermore, quercetin increased the scavenging ABTS+ ability. Overall, these findings provide a potential rationale for the application of quercetin in improving the EPS yield.

## 1. Introduction

*Monascus* is a non-pathogenic fungus which is widely used in food fermentation [1]. Derived by the fermentation of *Monascus*, it not only invigorates the spleen, promotes blood circulation, and removes blood stasis, but also reduces blood sugar, blood pressure, and lipids, and has anti-tumor effects [2]. Most studies on *Monascus* have focused on lovastatin, pigments, and cholesterol inhibitors. However, exopolysaccharides are becoming a research hotspot because of their rich functional activities and wide applications.

Exopolysaccharides (EPS) are important metabolites of *Monascus*. For example, red yeast rice exopolysaccharides extract can regulate immune activity [3] and has anti-tumor [4], anti-oxidation [5], and anti-inflammatory properties [6]. Similar to other exopolysaccharides from other fungi, *Monascus* exopolysaccharides can be produced through liquid fermentation. However, the production of EPS is generally low; therefore, there is some research on optimizing the yield of exopolysaccharides [7]. Wu et al. [8] optimized the liquid fermentation conditions of exopolysaccharides produced by *Monascus*, and the optimized exopolysaccharides’ yield could reach 999.8 mg/mL. Wang et al. [9] optimized the extraction conditions of intracellular exopolysaccharides through the Box–Behnken design.

The results showed that adding exogenous substances or optimizing fermentation conditions not only improves the secondary metabolites of fungi, but also significantly affects the structure and biological activity of fungal exopolysaccharides. Compared with the base medium, the optimized fermentation conditions not only improved *Cordyceps gunnii*’s molecular weight, but also significantly improved its anti-tumor effect on S180 cells [10]. By optimizing the culture medium of rice and soybean powder, Li et al. [11] increased the yield of exopolysaccharides by 1.3 times. Lu et al. [12] improved the production and antioxidant activity of EPS by Lentinula edodes by adding four different lignocellulose.

Our laboratory studied the effect of flavonoids on the reduction of citrinin production in *Monascus* fermentation, and explored the mechanism of the effect of flavonoids on citrinin reduction with the help of transcriptomics and H-1 NMR-based multivariate metabolomic analysis [13,14,15]. However, there is little information on the effect of the addition of flavonoids to the fermentation process on the quantity, structure, and antioxidant capacity of EPS produced by *Monascus*.

Therefore, this work aims to study the effect of flavonoids addition to the fermentation process on the amount, structure, and antioxidant capacity of EPS produced by *Monascus*.

## 2. Materials and Methods

### 2.1. Strains and Culture Medium

*Monascus purpureus* 30141 and *Monascus Anka* 30192 were obtained from the ACCC China Agricultural Microbial Species Preservation and Management Center. *Monascus purpureus* 40269 was obtained from the China Center of Industrial Culture Collection (CICC). *Monascus aurantiacus* Li AS3.4384 (*MAL*) was obtained from the China General Microbiological Culture Collection Center (CGMCC) (Beijing, China). The seed medium characteristics were as follows: 30 g/L glucose, 2 g/L KH_2_PO_4_, 3 g/L NaNO_3_, 0.5 g/L KCl, 0.5 g/L MgSO_4_·7H_2_O, and pH natural. The rice flour sodium nitrate liquid fermentation medium characteristics were as follows: 20 g/L rice flour, 2 g/L NaNO_3_, 0.5 g/L KH_2_PO_4_, 1 g/L K_2_HPO_4_·3H_2_O, 1 g/L MgSO_4_·7H_2_O, pH natural, and 5% inoculum. The sucrose yeast extract liquid fermentation medium characteristics were as follows: 50 g/L sucrose, 3.5 g/L yeast extract, 0.9 g/L KH_2_PO_4_, 1.8 g/L K_2_HPO_4_·3H_2_O, 1 g/L MgSO_4_·7H_2_O, 2 mL/L Tween-80, pH neutral, inoculum size 9%, shaking speed 180 r/min, and culture for 100 h.

### 2.2. Fermentation Process

Spore suspension (7 × 10^6^ spores/mL, 1 mL) was grown in a bottle at 30 °C for 48 h with constant shaking at 180 rpm. The seed solution (5 mL) was then inoculated onto the aseptic liquid fermentation medium (100 mL) and grown at 30 °C for 96 h under constant shaking at 180 rpm. EPS obtained from the culture containing different concentrations of quercetin were named Q-EMP and those obtained from the culture without quercetin were named EMP.

### 2.3. Determination of Crude EPS Yield and Biomass

After liquid fermentation, the fermentation liquid was filtered and centrifuged at 10,000g for 15 min. By rotating evaporation to the supernatant of the original volume of 1/4, 4 times the volume of anhydrous ethanol was added. The precipitation was centrifuged after 24 h at 4 °C and dried to constant weight. The weight conversion was calculated as the EPS yield. After vacuum freeze drying, the filtered mycelia were dried to a constant weight and weighed to obtain the biomass.

### 2.4. Determination of Extracellular Pigments

The fermentation broth and methanol were mixed in the ratio of 1:2, then water-bathed at 60 °C for 1 h. After the mixture was cooled and then filtered through a 0.22 μm organic filter membrane, the optical density (OD) was measured by an enzyme marker at 410 nm, 470 nm, and 510 nm. Using methanol as blank, the pigment content was calculated: yellow pigment = OD_410_ × dilution times (U/mL), orange pigment = OD_470_ × dilution times (U/mL), red pigment = OD_510_ × dilution times (U/mL).

### 2.5. Determination of Extracellular Citrinin

Briefly, 2.0 mL of methanol was added to 1.0 mL of fermentation broth and shaken in a 60 °C water bath for 1 h. After cooling, the fermentation broth was filtered through a 0.22 μm microporous organic membrane and detected via high-performance liquid chromatography (HPLC). The citrinin determination conditions were as follows: mobile phase ratio of acetonitrile to water, 60:40 (*v/v*); pH, approximately 2.5; flow rate, 1 mL/min; injection volume, 20 μL; excitation wavelength of fluorescence detector, 300 nm; and emission wavelength, 500 nm.

### 2.6. Optimization of Fermentation Medium Components

The medium components were optimized for carbon source type and carbon source concentration, nitrogen source type and nitrogen source concentration, carbon–nitrogen ratio, magnesium ion addition amount, potassium ion addition amount, surfactant type, and surfactant addition amount. In the process of medium composition optimization, the culture conditions were: pH 5.5, inoculum 5%, seed age 48 h, shaker speed 180 r/min, temperature 30 °C, incubation 96 h. Three parallel trials were conducted for each group.

### 2.7. Optimization of Fermentation Conditions

Based on the optimization of the medium components, the initial pH, inoculum size, seed age, shaking speed, fermentation time, and temperature after adding quercetin were optimized. Three parallel trials were conducted for each group.

### 2.8. Preliminary Characterization of Q-EMP

#### 2.8.1. Chemical Components Analysis

The neutral sugars, proteins, and glyoxylate contents of Q-EMP were determined by the phenol–sulfuric acid method, the koemas brilliant blue method, and the carbazole method, respectively [16].

#### 2.8.2. *Mw* Determination

The *Mw* of Q-EMP was determined using high-performance liquid chromatography (Agilent 1260) equipped with an Ultrahydrogel^TM^-1000 column (7.8 mm × 300 mm), as described in our previous report [17]. The flow rate of the sampling apparatus was programmed to 0.6 mL/min and the mobile phase was NaN_3_. T-series dextran (*Mw*: 10, 25, 40, 50, 70, 500, and 2000 kDa) was used as the standard to fit the linear regression curve. Finally, the *Mw* was calculated using the calibration curve equation based on the residence time of the Q-EMP peak.

#### 2.8.3. Monosaccharide Composition Analysis

The monosaccharide composition of Q-EMP was determined by high-performance anion exchange chromatography with pulsed amperometric detection (HPAEC-PAD), as previously described [17]. Briefly, 5 mg of Q-EMP was accurately weighed into a test tube containing H_2_SO_4_ solution (0.5 mL, 12 M) and magnetically stirred in an ice bath for 30 min. Immediately afterwards, distilled water (2.5 mL) was added to the test tube and the reaction was carried out in an oil heat bath at 110 °C for 2.5 h. After the reaction was completed, it was cooled and the reaction solution was diluted by a certain number of times. Finally, the solution was filtered through a 0.22 μm water membrane and injected. The standard curve was plotted from 10 monosaccharide specimens, including glucose (Glc), rhamnose (Rha), xylose (Xyl), galactose (Gal), arabinose (Ara), fructose (Fru), fucose (Fuc), mannose (Man), glucuronide (GlcA), and galacturonic acid (GalA).

### 2.9. Determination of Antioxidant Capacity

#### 2.9.1. Determination of DPPH·Scavenging Capacity

The ability of EPS extract to scavenge 2,2-diphenyl-1-picrylhydrazyl (DPPH) free radicals was determined by referring to the method of Mohsen et al. [18], with a slight modification. The experiments were prepared with EPS sample solution according to concentration gradients of 0.1 mg/mL, 0.4 mg/mL, 0.8 mg/mL, 1.2 mg/mL, 1.6 mg/mL, and 2.0 mg/mL. The reaction mixtures contained 2 mL of DPPH solution, 1.5 mL of deionized water, and 0.5 mL of the corresponding concentration of EPS solution. After being placed in a dark environment for 0.5 h, the OD_517_ value was measured with a microplate reader. *Vitamine C* (Vc) was used as a positive control and 95% ethanol was used as a negative control. The formula for the DPPH scavenging effect is as follows:

(1) $$\mathrm{DPPH}\cdot \;\mathrm{scavenging}\;\mathrm{rate}\;\left(\%\right)=\left[A_{0}-\left(A_{2}-A_{1}\right)\right]/A_{0}\times 100\%,$$

where *A*_0_ is 2 mL DPPH + 2 mL water OD_517_, *A*_1_ is 2 mL ethanol + 0.5 mL EPS solution + 1.5 mL water OD_517_, and A_2_ is 2 mL DPPH + 0.5 mL EPS + 1.5 mL water OD_517_.

The percentage of scavenging activity was plotted against the concentration of the sample to obtain the half maximal inhibitory concentration (IC_50_), which was defined as the concentration of the sample required to cause 50% inhibition. 

#### 2.9.2. Determination of -OH Scavenging Capacity

The scavenging activity of the extract on hydroxyl radicals was determined using the method of Smirnoff et al. [19]. The reaction mixtures contained 1 mL of the sample solution, 1 mL of prepared FeSO_4_, 1 mL of salicylic acid solution, and 1 mL of H_2_O_2_ solution. The solution was mixed thoroughly and placed in a water bath at 37 °C for 0.5 h. Vc was used as a positive control. The formula for calculating the -OH· scavenging activity is as follows:

(2) $$-\mathrm{OH}\;\mathrm{scavenging}\;\mathrm{rate}\left(\%\right)=\left[1-\left(A_{2}-A_{1}\right){/A}_{0}\right]\times 100\%,$$

where *A*_2_ is 1 mL FeSO_4_ + 1 mL salicylic acid–ethanol solution + 1 mL EPS solution + 1 mL H_2_O_2_ OD_510_, *A*_1_ is 1 mL FeSO_4_ + 1 mL salicylic acid–ethanol solution + 1 mL EPS solution + 1 mL H_2_O OD_510_, and *A*_0_ is OD_510_ of 1 mL FeSO_4_ + 1 mL salicylic acid–ethanol solution + 1 mL H_2_O + 1 mL H_2_O_2_.

#### 2.9.3. Determination of ABTS^+^ Scavenging Ability

The 2,2′-Azinobis-(3-ethylbenzthiazoline-6-sulphonate) (ABTS^+^) radical scavenging activity is a manifestation of the hydrogen supply capacity and chain-scission ability of the sample [20]. The scavenging activity of the active extract on 2,2′-diaza-bis (3-ethylbenzothiazole-6-sulfonic acid) ammonium salt (ABTS) free radicals was determined by referring to the method of Re et al. [21] after modification.

The ABTS^+^ radical reaction solution configuration was as follows: 5 mL of 7 mmol/L of ABTS and 5 mL of 2.45 mmol/L of potassium persulfate were mixed and stored in the dark for 12 h. Before use, 0.1 mol/L of pH 7.4 phosphate buffer saline (PBS) was added to dilute until the OD_734_ value was 0.70 ± 0.02. The sample solution was the same as that of the EPS sample solution measured by DPPH clearing ability. The reaction mixture contained 40 μL of the sample solution and 970 μL of the reaction solution. Afterward, the mixtures were reacted in the dark for 6 min and the OD_734_ value was measured using a microplate reader. The formula for calculating the ABTS^+^ scavenging effect is calculated as follows:

(3) $${\mathrm{ABTS}}^{+}\cdot \;\mathrm{scavenging}\;\mathrm{rate}\left(\%\right)=\left(1-A_{1}{/A}_{0}\right)\times 100\%,$$

where *A*_0_ is the ABTS^+^ free radical reaction solution and *A*_1_ is the sample group.

### 2.10. Data Analysis

Data were expressed as the mean ± standard deviation (SD) of three replicates. SPSS 19.0 (IBM, Armonk, NY, USA) was used to analyze significant differences between the two groups, with *p* < 0.05 differences considered statistically significant.

## 3. Results

### 3.1. Effects of Species of Fungus on EPS Production

**Figure 1:**
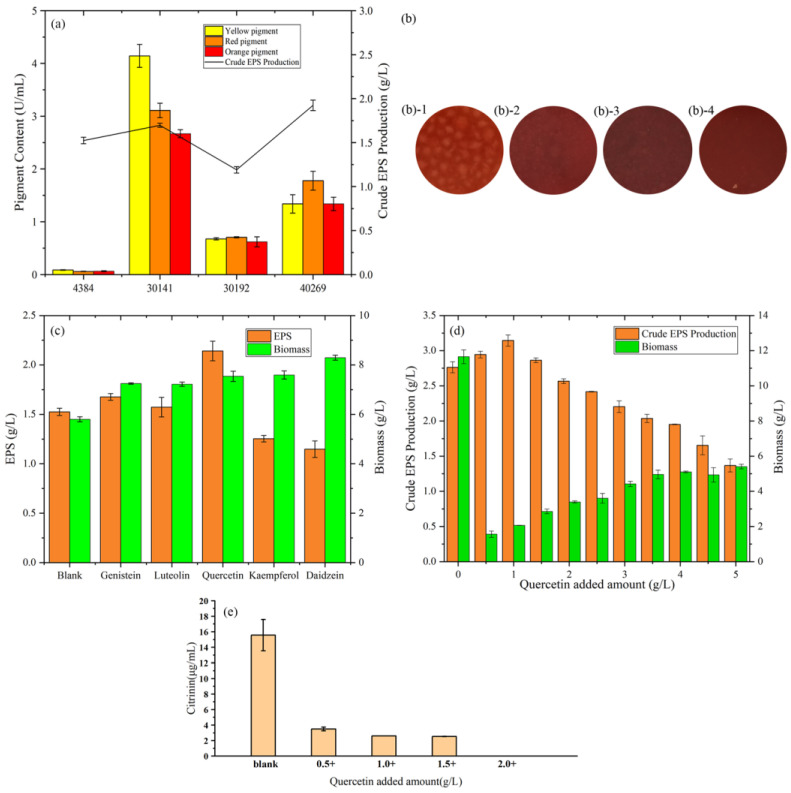
(**a**) Comparison of the extracellular pigment and crude EPS changes of four *Monascus* strains. (**b**) Growth diagram of four strains of *Monascus*: (**b-1**) represents *Monascus aurantiacus* AS3.4384, (**b-2**) represents *Monascus purpureus* 30141, (**b-3**) represents *Monascus anka* 30192, (**b-4**) represents *Monascus purpureus* 40269. (**c**) Effects of the addition of flavonoids to *Monascus aurantiacus* AS3.4384 on the production of crude EPS and biomass. (**d**) Effect of different addition of quercetin on *Monascus aurantium* AS3.4384 on the production of crude EPS and biomass. (**e**) The production of citrinin after adding quercetin to *Monascus aurantium* AS3.4384 in the sucrose yeast extract medium for 4 days.

According to Figure 1a, among the four strains, *M. purpureus* 40269 had the highest. EPS yield. From the view of EPS production, low pigment production is beneficial to the subsequent purification, which is helpful to study the physical and chemical properties of EPS. After 4 days of fermentation, the extracellular pigment production capacity of the blank group is as follows: *M. purpureus* 30141 > *M. purpureus* 40269 > *M. anka* 30192 > *MAL*. It can also be seen from the growth figures (Figure 1b) after fermentation that the pigment production of *MAL* was the lowest. Therefore, *MAL* was selected as the strain for subsequent crude EPS yield optimization.

### 3.2. Effects of Flavonoids on crude EPS Yield

From Figure 1c, compared with 1.52 g/L in the blank group, quercetin increased the EPS yield to 2.14 g/L, which was 40.8% higher than the blank group. The addition of genistein and luteolin increased the yield of EPS to 1.68 g/L and 1.57 g/L, respectively, by 10.5% and 3.5%. Furthermore, it is different from the results of Xie’s study [22] and may be due to the different fermentation strains. Genistein can promote EPS production by regulating membrane permeability, enhancing cell respiration metabolism and monosaccharide precursor synthetase activity, which provides direction for exploring the mechanism of quercetin to increase exopolysaccharide yield [23]. The addition of kaempferol and daidzein reduced the EPS yield to 1.25 g/L and 1.15 g/L, respectively, which decreased by 17.8% and 24.3%. From the point of view of biomass, the addition of flavonoids can increase the biomass, which may be part of the unused flavonoids attached to the fungi surface after fermentation.

### 3.3. Effects of Quercetin Addition on M. purpureus Biomass and EPS Yield

Results of the effect of quercetin addition on *M. purpureus* biomass and EPS yield after 96 h of liquid fermentation are shown in Figure 1d. *M. purpureus* biomass increased with an increase in quercetin concentration (from 1 g/L to 5 g/L), peaking at 5 g/L, indicating that quercetin significantly affected *M. purpureus* biomass. Moreover, the addition of quercetin enhanced the secretion of EPS by *M. purpureus*, with the highest EPS yield (3.14 g/L) obtained at a concentration of 1.0 g/L quercetin. In summary, the most suitable amount of quercetin to obtain the maximum amount of EPS was determined to be 1.0 g/L.

*MAL* has a high yield of toxic by-product, citrinin. Therefore, it is necessary to detect citrinin during fermentation. As shown in Figure 1e, after being fermented in sucrose yeast powder medium for 4 days, the yield of citrinin in the blank group was 15.56 μg/mL. The yield of citrinin gradually decreased with the increased addition of quercetin, and no citrinin could be detected when the addition reached 2 g/L. The results showed that *Monascus* EPS could eliminate the citrinin residual problem.

### 3.4. Effect of Culture Medium on EPS-Production

#### 3.4.1. Effect of Carbon Source Type and Carbon Source Concentration 

**Figure 2:**
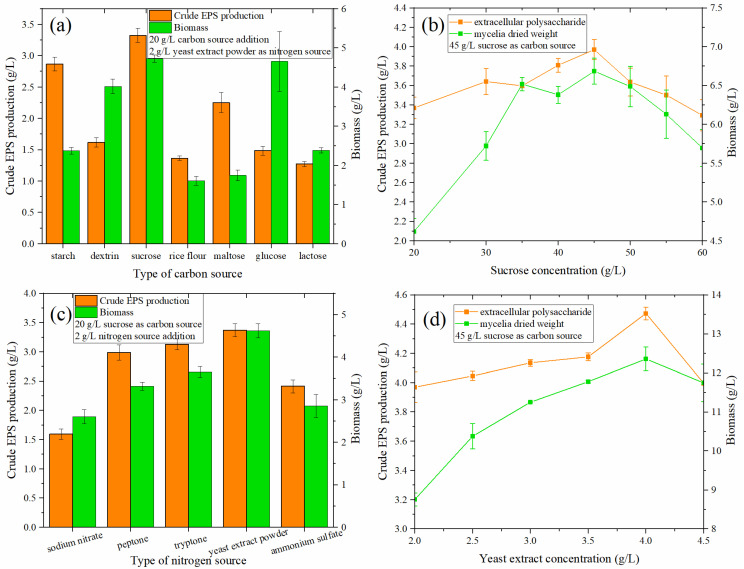
(**a**) Effect of carbon source type on the crude EPS production and biomass of *MAL*. (**b**) Effect of sucrose concentration on crude EPS and biomass produced by *MAL*. (**c**) Effect of nitrogen source type on the crude EPS production and biomass of *MAL*. (**d**) Influence of yeast extract concentration on the crude EPS and biomass produced by *MAL*.

The type of carbon source can affect the growth of microorganisms and the secretion of secondary metabolites. Seven different carbon sources (20 g/L)—rice flour, sucrose, glucose, maltose, lactose, dextrin, and water-soluble starch—were selected. The optimization results for the carbon source types in the medium are shown in Figure 2a. When the amount of the carbon source added was 20 g/L, sucrose had the greatest effect on the production of crude EPS, reaching 3.324 g/L. Water-soluble starch had an amount of 2.866 g/L, followed by maltose at 2.252 g/L. 

In terms of the ability to produce EPS and promote the growth of fungi, sucrose is superior to other carbon sources. At the same time, sucrose can also avoid the cloudy fermentation broth caused by rice flour. Moreover, the problem of EPS residues is mitigated. This is also consistent with some reports that sucrose was the most suitable carbon source for biomass and EPS production [24]. 

When the sucrose content increased from 20 to 60 g/L (Figure 2b), the crude EPS exhibited a trend of first increasing and then decreasing, reaching a maximum value of 3.969 g/L at 45 g/L. Compared with 3.368 g/L at 20 g/L, the output value of crude EPS increased by 17.84%. Simultaneously, the biomass of the fungi also exhibited the same trend as the crude EPS as the concentration of the carbon source increased.

#### 3.4.2. Effect of Nitrogen Source Type and Nitrogen Source Concentration

Sodium nitrate, peptone, tryptone, yeast extract powder, and ammonium sulfate were used as nitrogen sources in the liquid fermentation medium. When the amount of nitrogen source was 2 g/L (Figure 2c), the ability of yeast extract powder to produce EPS was the strongest, reaching 3.368 g/L, followed by tryptone (3.126 g/L), peptone (3.0 g/L), ammonium sulfate (2.412 g/L), and sodium nitrate (1.592 g/L). From the perspective of cell growth (Figure 2d), yeast extract powder exhibited the best growth, followed by tryptone, peptone, ammonium sulfate, and sodium nitrate. Powder was the optimal nitrogen source. This is consistent with the results of previous studies, e.g., [25,26], which revealed that organic nitrogen is more effective than inorganic nitrogen in the utilization of *Monascus* nitrogen sources. 

From Figure 2d, it can be found that when the yeast extract concentration is 4.0 g/L, the EPS production capacity of *Monascus* was the highest (4.473 g/L), increasing by 32.77% compared with that at the initial addition of 2.0 g/L (3.969 g/L); similarly, cell growth was also highest under these conditions.

#### 3.4.3. Effect of Carbon–Nitrogen Ratio

The carbon–nitrogen ratio was varied by changing the ratio of sucrose and yeast extract according to Table 1. If the carbon–nitrogen ratio is too high or too low, it will have adverse effects on cell growth, foreign protein expression, and accumulation [27]. As shown in Figure 2e, the EPS production first decreased and then increased with increased yeast extract concentration. When the C/N ratio was 78:1, the crude EPS content was 4.753 g/L, which was 4.473 (or 6.26%) g/L higher than that under a C/N ratio of 61:1. This is consistent with the findings of Mian et al. [28], where the specific EPS production increased with a lower nitrogen concentration. 

The carbon-to-nitrogen ratio is calculated as (sucrose content × carbon content of sucrose)/(yeast extract content × nitrogen content of yeast extract), where carbon content of sucrose is defined as (the sum of relative atomic mass of carbon atoms in a sucrose molecule)/(the relative molecular mass of sucrose) and the nitrogen content of yeast extract is calculated in a similar way.

#### 3.4.4. Effect of Inorganic Salt Ions

Inorganic salts are one of the six nutrient elements required for the growth of microorganisms. Their concentration has a significant impact on the growth of fungi cells and the formation of the *Monascus* pigment [29], and they provide the necessary elements or precursors for the formation of fermentation metabolites [30]. Mg^2+^ is an activator of many cytochrome enzymes [31] and S is a component of physiologically active substances. Therefore, the addition of MgSO_4_ to the culture medium may have an effect on fungi growth and the accumulation of secondary metabolites. Studies have revealed that the secondary metabolic pathway of *Monascus* contains phosphate but the final product does not, indicating that phosphatase is likely to participate in biosynthesis [32]. At the same time, K^+^ may be a cofactor in some of the reactions in the exopolysaccharides’ synthesis process [33]. Hence, KH_2_PO_4_ and K_2_HPO_4_·3H_2_O added to the medium can achieve feedback and inhibition effects.

**Figure 3:**
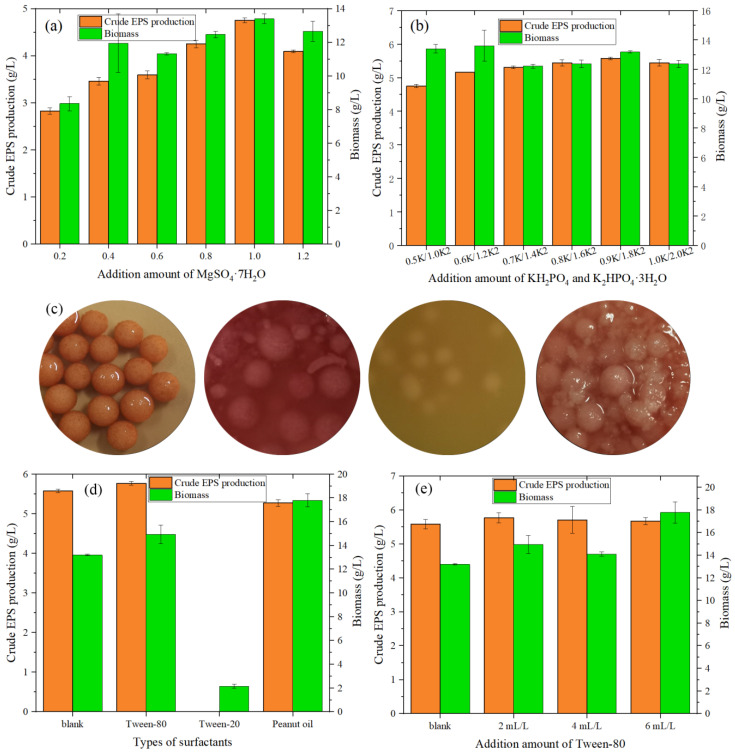
(**a**) Effect of MgSO_4_·7H_2_O on the crude EPS production and biomass of *MAL*. (**b**) Effect of KH_2_PO_4_ and K_2_HPO_4_·3H_2_O on the EPS and biomass of *MAL*. (**c**) Liquid culture medium of *MAL* with different surfactants (from left to right: blanks, with Tween-80, with Tween-20, and with peanut oil). (**d**) Effect of surfactant type on the crude EPS production and biomass produced by *MAL*. (**e**) Effect of Tween-80 addition on the EPS and biomass of *MAL*.

From Figure 3a, when the addition amount of MgSO_4_·7H_2_O increased from 0.2 to 1.2 g/L, the value of crude exopolysaccharide increased, reaching a maximum (4.753 g/L) when the addition amount was 1.0 g/L. At this time, the biomass of the fungi also reached the optimal value. From Figure 3b, when the addition amount of KH_2_PO_4_ was 0.9 g/L and the addition amount of K_2_HPO_4_·3H_2_O was 1.8 g/L, the value of crude EPS peaked at 5.576 g/L, 17.32% higher than when the initial KH_2_PO_4_ was added at 0.5 g/L and K_2_HPO_4_·3H_2_O was added at 1.0 g/L (crude EPS of 4.753 g/L). 

#### 3.4.5. Effect of Type and Content Optimization of Surfactants

Park et al. [34] revealed that surfactants can increase the permeability of cell membranes and improve the oxygen supply and transport capacity, thereby affecting the accumulation of secondary metabolites.

As shown in Figure 3d, compared with the blank group (5.576 g/L), the addition of Tween-80 increased the value of crude EPS to 5.767 g/L, representing in an increase of 3.43%. The addition of peanut oil reduced the value of crude EPS to 5.27 g /L, which may be related to the decrease in the volume of the fermentation broth after vigorous growth of the fungi, leading to a decrease in the value of the EPS. The addition of Tween-20 inhibited the growth of *Monascus* while the fungi were small and the color did not change after 4 days of fermentation.

After optimizing the amount of addition, in Figure 3e it can been seen that multi-dose addition promoted the growth of fungi but was not conducive to the accumulation of EPS. When surfactant addition is extremely low, both the growth of fungi and the production of EPS are inhibited. Furthermore, an addition of 2 mL/L was sufficient to promote the production of EPS (production value of 5.767 g/L), with the growth of fungi being slightly better than that of the blank. 

### 3.5. Effect of Cultivation Conditions on EPS Production

#### 3.5.1. Effect of Initial pH

pH is an important factor that determines microbial growth and metabolic activity [35]. *Monascus* is suitable for growth in weakly acidic environments, particularly lactic acid. 

**Figure 4:**
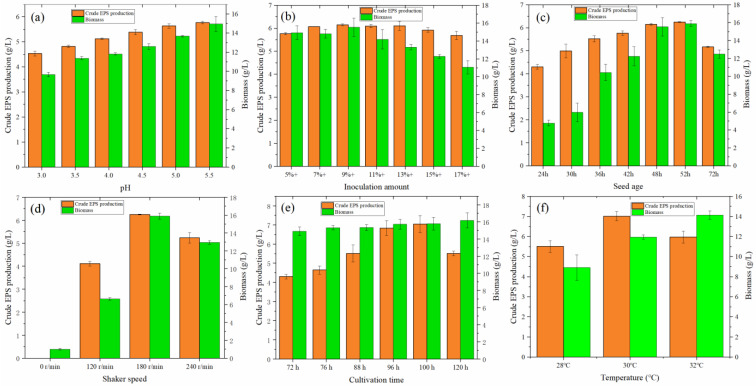
(**a**) Effect of initial pH on the production of crude EPS and biomass of *MAL.* (**b**) Effect of inoculation amount on the crude EPS and biomass produced by *MAL.* (**c**) Effect of seed age on the crude EPS and biomass of *MAL.* (**d**) Effect of shaker speed on the crude EPS and biomass produced by *MAL.* (**e**) Effect of culture time on the crude EPS and biomass produced by *MAL*. (**f**) Effect of temperature on the crude EPS and biomass produced by *MAL*.

From Figure 4a, with increasing pH value in the range of 3.0–5.5, the yield of crude EPS and cell growth both increased. At pH 5.5, the crude EPS reached an optimal value of 5.767 g/L, and cell growth was also good. This is partially inconsistent with the experimental results of Li et al. [11], Zeng et al. [26], and Wu [8]. Wu [8] revealed that although *Monascus* N grew well at pH < 4.5, these conditions were not conducive to the accumulation of exopolysaccharides. When the pH was 5.5, the concentration of *Monascus* exopolysaccharides reached 890.4 mg/L. Li et al. [11] added lactic acid to adjust the effect of pH on the production of crude EPS. With the increase in pH from 3.0 to 5.0, the production of crude EPS showed a trend of slow increase, but it was lower than that of natural conditions. Wang et al. [9] used *Monascus* Mr70 as the research object and revealed that with an increase in pH from 3.5 to 7.0, the yield of crude EPS also increased, reaching a maximum at pH 6.5; however, the biomass of fungi gradually decreased. They concluded that acidic conditions are conducive to the growth of fungi but not to the accumulation of exopolysaccharides.

In this study, the strain *Monascus aurantiacus* was not conducive to cell growth or EPS accumulation under strongly acidic conditions. It can be speculated that the optimal growth range of this strain is in a weakly acidic environment, which is consistent with the growth of *Aspergillus* in weakly acidic environments [36].

#### 3.5.2. Effect of Inoculation Quantity

The size of the inoculum is a decisive factor in the growth and reproduction of fungi [37,38]. From Figure 4b, when the inoculation amount gradually increased from 5% to 17%, the yield of crude EPS first increased and then decreased. The yield reached a maximum value of 6.149 g/L when the inoculation amount was 9%, which was 6.62% higher than that of the initial inoculation amount of 5%. It can be observed from the biomass that the cell growth exhibited a trend of high-to-low growth with the increase in inoculation amount, indicating that inoculation amount is closely related to cell growth. Moreover, more independent mycelia could secrete more EPS.

#### 3.5.3. Effect of Age Optimization 

As shown in Figure 4c, when the seed age gradually increased from 24 to 72 h, the yield of crude EPS first increased and then decreased. When the seed age was 52 h, the EPS yield was slightly higher than that at 48 h. From the perspective of EPS production and time, 52 h is a better choice.

#### 3.5.4. Effect of Shaker Speed

*Monascus* is an aerobic fungus [39]. Under liquid fermentation conditions, oxygen content can be increased by increasing the speed of the shaker. As shown in Figure 4d, when the rotation speed was 0 r/min, no evident fungi balls were observed. The fermentation broth did not change color, indicating that the static culture was not conducive to the liquid fermentation of *Monascus*. When the rotation speed was gradually increased from 0 r to 240 r/min, the crude EPS and cell biomass of *Monascus* increased at first and then decreased. At 180 r/min, the fungi grew better, and the value of exopolysaccharide secreted was 6.250 g/L. At 240 r/min, the fermentation broth had a darker color; however, the amount of fungi was reduced, indicating that high speed is also not conducive to EPS production.

#### 3.5.5. Effect of Culture Time

As shown in Figure 4e, the secretion of crude EPS reached a maximum value of 6.285 g/L at 100 h, which was 0.56% higher than that at 96 h. The fungus grew logarithmically in the pre-fermentation period, metabolites accumulated in the middle and late stages, and the fungus gradually senesced as the growth stabilized. The production of EPS in fermentation broth also verified this law. Before 100 h, the yield increased gradually with the increase in fermentation time. After 100 h, the EPS yield started to decrease, suggesting that the lack of nutrients for the growth of the fungus causes the consumption of EPS more than the yield. The results indicated that the optimal fermentation time was 100 h.

#### 3.5.6. Effect of Culture Temperature after Adding Quercetin

Based on the above optimization conditions, 1.0 g/L quercetin was added to the liquid medium. As shown in Figure 4f, it was found that the production of EPS first increased and then decreased. At 30 °C, the production of EPS reached its maximum (7.018 g/L). It also proved that the initial experimental setting temperature was the appropriate temperature. The addition of quercetin increased the production of EPS by 11.66%.

### 3.6. Physicochemical Properties

The chemical compositions and yields of Q-EMP and EMP are shown in Table 2. Compared with EMP, there was a significant increase in neutral sugar and a decrease in the protein content and uronic acid content of Q-EMP. These results suggest that the addition of quercetin can promote the exudation of several chemical moieties. Table 2 shows that the *Mw* of Q-EMP (52.104 kDa) is significantly lower than that of EMP (166.864 kDa). Previous studies have shown that the bioactivity of EPS seemed to be affected by its molecular weight [40]. The EPS with low molecular weight seem to be more effective for antioxidant activity [41,42,43]. Therefore, it can be estimated that the relatively lower molecular weight of Q-EMP may be helpful for its antioxidant activity.

### 3.7. Monosaccharide Composition

**Figure 5:**
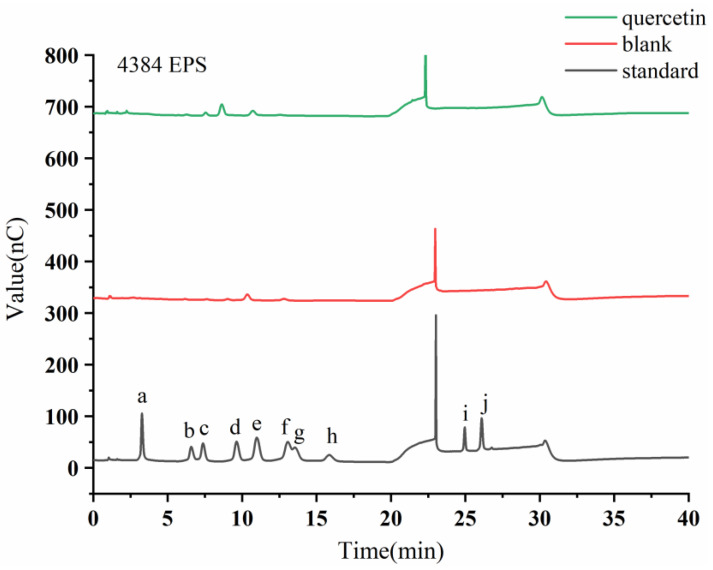
HPAEC-PAD chromatogram profiles of extracellular *Monascus* exopolysaccharides after adding flavonoids. The letters a-j denote the monosaccharide standards of Fuc, Rha, Ara, Gal, Glc, Xyl, Man, Fru, GalA, and GlcA, respectively.

HPAEC-PAD was used to determine the monosaccharide compositions of Q-EMP and EMP, which are closely related to their bioactive characteristics [44]. From Figure 5, it can be found that the monosaccharide composition of EPS changed significantly after the addition of quercetin. Table 2 demonstrates that QMP is primarily composed of Fru, Gal, Glu, and Xyl at a molar ratio of 1:1.3:33.8:13.2 after fermentation, which is consistent with the monosaccharide species in the study by Wang et al. [45]. The molar percentages of Glu and Xyl significantly increased after fermentation. These results suggest that the addition of exogenous substances potentially change the proportion of monosaccharides. 

### 3.8. Determination of Antioxidant Capacity

#### 3.8.1. DPPH Scavenging Activity

**Figure 6:**
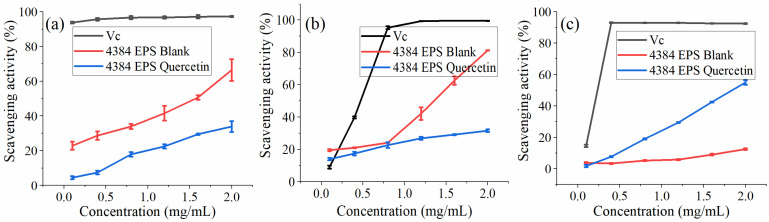
(**a**) Ability of EPS and Q-EMP to scavenge DPPH (2,2-diphenyl-1- picrylhydrazyl) free radicals. (**b**) Ability of EPS and Q-EMP to scavenge OH free radicals. (**c**) Ability of EPS and Q-EMP to scavenge ABTS free radicals.

The DPPH test is used to evaluate the free radical scavenging ability of specific substances and is widely used in food biochemistry and plants [46]. From Figure 6a, when the concentration of EPS increased from 0.1 to 2.0 mg/mL, the ability of the blank group to clear DPPH increased from 22.80% to 66.38%, which is consistent with the conclusion that the ability to clear DPPH is concentration-dependent [11,47,48]. The ability to remove DPPH in the quercetin group increased from 4.4% to 33.82%. Compared with the blank group, the addition of quercetin reduced its scavenging DPPH ability.

#### 3.8.2. -OH Scavenging Capacity

The hydroxyl radical easily reacts with neighboring cells or biological molecules and causes serious oxidative damage to cells or biological molecules, leading to diseases such as aging and cancer [49]. From Figure 6b, when the concentration of EPS increased from 0.1 to 2.0 mg/mL, the ability of the blank group to eliminate -OH increased from 19.47% to 81.15%. IC_50_ was approximately 1.4 mg/mL. The ability to remove -OH in the quercetin group increased from 13.92% to 31.51%. The scavenging -OH ability at the lowest concentration of 0.1 mg/mL is significantly better than the experimental group added with quercetin.

#### 3.8.3. ABTS^+^ Scavenging Ability

ABTS radical cations are scavenged by providing hydrogen to form stable ABTS molecules [50]. This can reflect the antioxidant capacity of the determination samples [51]. From Figure 6c, when the EPS concentration increased from 0.1 to 2.0 mg/mL, the scavenging ABTS^+^·ability of the blank group increased from 3.75% to 12.44%. The scavenging activity of EPS-Quercetin exhibited a concentration-dependent manner and was much higher than that of the blank group at the same concentrations, while the ability to remove ABTS+ increased from 1.66% to 54.88%, and the IC_50_ value was close to 1.9 mg/mL. It showed that *Monascus* exopolysaccharides had a certain ABTS radical scavenging ability and showed a slight concentration dependence. This result is consistent with the study of Wang et al. [52].

## 4. Conclusions

In this study, the strains of *Monascus* exopolysaccharides’ fermentation were optimized, and it was found that quercetin had the best effect on EPS yield improvement. By optimizing the composition of the medium and the culture conditions, the best method for the production of EPS by *Monascus aurantiacus* AS3.4384 was obtained as follows. Sucrose yeast extract liquid fermentation medium: 50 g/L sucrose, 3.5 g/L yeast extract powder, 1.0 g/L Mg SO_4_·7H_2_O, 0.9 g/L KH_2_PO_4_, 1.8 g/L K_2_HPO_4_·3H_2_O, 2 mL/L Tween-80, 1 g/L quercetin, pH of 5.5, inoculum amount of 9%, seeding age of 52 h, and cultured for 100 h at a shaker speed of 180 rpm. Under these conditions, the fermented crude extracellular exopolysaccharide value reached its maximum, and the output was 7.018 g/L. In addition, quercetin increased the yield of EPS by 11.66%. Structural studies showed that quercetin addition significantly affected neutral sugar, protein, uronic acid, and *Mw*, and molar ratios of monosaccharides of EPS produced by *Monascus aurantiacus*. It was found that *Monascus* exopolysaccharides have good scavenging effects on DPPH and OH, and the addition of quercetin also improved the scavenging ability of ABTS. These results show that quercetin addition to the fermentation process can increase the yield of *Monascus aurantiacus* exopolysaccharides, and even improve their antioxidant ability activity. However, further work is needed to explore the mechanisms by which exogenous quercetin affects the secretion and structure of *Monascus aurantiacus* exopolysaccharides.

## Figures and Tables

**Table 1:** Carbon-to-nitrogen ratio corresponding to sucrose and yeast extract content, and the yield of crude extracellular exopolysaccharide and biomass under corresponding conditions.

Carbon-to-Nitrogen Ratio	Sucrose Content (g/L)	Yeast Extract Content (g/L)	Crude EPS Production (g/L)	Biomass (g/L)
30:1	30	5.5	2.37 ± 0.02	7.73 ± 0.07
38:1	35	5	2.86 ± 0.03	8.22 ± 0.20
49:1	40	4.5	3.41 ± 0.18	10.61 ± 0.10
61:1	45	4	4.47 ± 0.04	11.23 ± 0.90
78:1	50	3.5	4.75 ± 0.05	9.85 ± 0.02
100:1	55	3.0	3.89 ± 0.04	11.45 ± 0.29

**Table 2:** Chemical composition of G-EMP.

Samples		4384 EPS Blank	4384 EPS Quercetin
Change in neutral sugar (%)		34.69 ± 0.41	37.73 ± 0.78
Change in uronic acid (%)		19.38 ± 0.34	8.87 ± 0.78
Change in protein (%)		3.01 ± 1.66	2.96 ± 0.50
Mw (kDa)		166.864	52.104 *
Monosaccharide composition (mole %) ^1^	Ara	0.6 ± 0.02	
	Fru	1.8 ± 0.15	0.6 ± 0.01 *
	Gal	1.8 ± 0.09	0.80 ± 0.2 *
	GalA		
	Glu	6.7 ± 0.07	20.3 ± 1.28 *
	Xyl	0.8 ± 0.02	7.9 ± 0.71 *
Mole Ratio		Ara:Fru:Gal:Glu:Xyl = 1:3:3:11.2:1.3	Fru:Gal:Glu:Xyl = 1:1.3:33.8:13.2

Data represent mean ± SD (n = 3). * *p* < 0.05 compared with EMP. ^1^ Ara: arabinose; Fru: fructose; Gal: galactose; Glu: glucose; GalA: galacturonic acid; Xyl: xylose.

## Data Availability

The data that support the findings of this study are available from the corresponding author upon reasonable request.
